# Substantiating Nexus Between Consumption Values and Sustainable Consumption Behavior: A Way Toward Sustainable Business

**DOI:** 10.3389/fpsyg.2022.908391

**Published:** 2022-06-09

**Authors:** Jianmin Sun, Huma Safdar, Zain ul Abidin Jaffri, Syed Ibn-ul-Hassan, Ilknur Ozturk

**Affiliations:** ^1^School of Management, Nanjing University of Posts and Telecommunications, Nanjing, China; ^2^Department of Management Sciences, COMSATS University Islamabad, Abbottabad, Pakistan; ^3^College of Physics and Electronic Information Engineering, Neijiang Normal University, Neijiang, China; ^4^Department of Commerce and Business, Government College University Faisalabad, Layyah, Pakistan; ^5^Faculty of Economics, Administrative and Social Sciences, Nisantasi University, Istanbul, Turkey

**Keywords:** sustainable consumption behavior, green products, consumption values, sustainability, environmental management

## Abstract

The unprecedented economic growth in recent decades has cultivated the exploitation of natural resources and over-consumption, leading to ecological deterioration and sustainability. The ever-increasing consumption in developing countries is creating a significant environmental strain. Thus, the industry and consumers’ environmental issues and their harmful effects on human health have led to concerns among researchers, scientists, academic communities, and policymakers. The present work examines the impact of different consumption value factors on sustainable consumption behavior concerning consumer choice in Pakistan and China. A cross-sectional study is conducted, and data are collected through a primary source questionnaire. A sample of 431 respondents is chosen from different cities in Pakistan, and a sample of 342 respondents is selected from China. Estimation techniques like descriptive statistics, frequency distribution, multicollinearity, *R* square, independent sample *t*-test, the coefficient of correlation, and regression analysis are used for the data analysis. The comparative results show that knowledge values (KVs) and emotional values (EMVs) significantly influence the choice behavior of respondents toward environmentally friendly products both in Pakistan and China. In contrast, social values (SVs) and conditional values (CVs) show insignificant influence. Furthermore, functional values (FVs) are significant in Pakistan while insignificant in the context of China, and environmental values (EVs) are significant in China although insignificant in Pakistan with regard to sustainable consumption behavior.

## Introduction

Over recent decades, increasing globalization and industrialization have drastically influenced the world’s climate, thus prompting calls for nature’s preservation. History indicates that, with the accelerating atmospheric emissions, human civilization has crossed the consumption boundary, thereby requiring the management of environmental consequences. However, in this regard, sustainable consumption has emerged as a novel solution to mitigating climate change. This noble 21st century development has shifted the consumers’ focus to organic products (e.g., eco-friendly, natural, and renewable), substantially elevating the concept of green buying behavior. This eco-friendly approach has altered the individual’s buying behavior, making sustainable consumption the most relevant research topic influencing consumer behavior (Brach et al., 2018).

In particular, several approaches have developed, and numerous theories have been proposed to characterize sustainable consumption. However, little attention is paid to consumers’ behavior changes despite awareness that consumers’ interests in product sustainability and green products and services have rapidly increased in recent years. In the current socio-political environment, green conservation has gained prevalence because of concerns about destroying Earth’s natural resources (Pino et al., 2012). Today, users and consumers are inspired to play a necessary part in developing growth through green preservation approaches (Moser, 2015). Therefore, consumer behavior is trending toward green products and services, which is also quite helpful in the reduction of solid waste (Ramayah et al., 2010).

In modern society, healthier living demands that humans should consume eco-friendly products. This permanent requirement has considerably built the pressure for adopting green consumption. Green or eco-friendly products and service choices are a way to decrease harmful environmental effects (Leonidou et al., 2013). Some alternate terminologies in use for the “Green” concept are “Sustainable,” “Eco-friendly,” “Ecologically Sound,” or “Environment-friendly” (Kim et al., 2013). The concept of green consumption first emerged as an approach in the 1970s (Peattie, 2010).

However, today’s climate change crisis has directed the consumers’ behavior more strongly toward green consumption than ever before. Environmental issues and concerns have caused a notable increase in buyers’ requirements and expectations for green products and services. This concern for the environment has made consumers focus on sustainable consumption, triggering sustainable buying behavior (Rusyani et al., 2021). Simultaneously, promoting green sustainability has made organizations recognize consumers’ green behavior as the most identified market demand. This demand has perhaps been generated by up-to-date ecological beliefs, which have improved people’s understanding and remarkably changed their behavior choices (Jang et al., 2011). The prioritizing process within customers’ behavior has created an association between the environment and the attributes of the green items. These items are further related by eco-friendly product utilization instead of special promotion practice (Schuhwerk and Lefkoff-Hagius, 1995).

A lot of research has been done previously regarding green consumption behavior. The main aim behind these studies was to identify the determinants that influence green behavior. The determinants include demographics (Diamantopoulos et al., 2003), values, environmental knowledge (Ramayah et al., 2010), attitudes (Chan, 2001), and internal and external moderators (Rylander and Allen, 2001). In addition, the research on green consumption also combines the existing models and theories, which are mainly supported by Reasoned Action Theory (Ajzen and Fishbein, 1980) and Planned Behavior Theory (Ajzen, 1991).

Environmental issues regarding product consumption and their harmful effects on human health have led to concerns among researchers, the academic community, policymakers, and firms. Environmental sustainability has emerged as a shift in attitudes toward response, which subsequently transformed user consumer behavior and their demand (Mendelson et al., 1996). Buyers have increasingly shown interest in sustainable goods and services. Also, comparably, concerns and knowledge about these issues have created genuine interest in “green consumerism” (Moisander, 2007).

The developed countries have increasingly moved toward green products, services, and consumption. At the same time, the growth toward green acceptance can also be examined among buyers in developing countries, for example, India (Raghavan et al., 2008). Research regarding environmental concerns and eco-friendly purchasing has been conducted in the Indian setting, contrasting with other developing countries (Khare, 2015). Indian businesses, educationists, and scholars are primarily focused on establishing appropriate plans and strategies for eco-friendly products and services. Yet, in prior research, various studies on consumer behavior and intention to buy green items have typically been undertaken within the framework of advanced countries (Kim et al., 2013). Some research (Biswas and Roy, 2015; Khare, 2015) works have considered buyers’ reactions toward green items in the Indian context, keeping in mind that India is a developing country. However, in our understanding, no studies in developing countries have measured customers’ goals (with particular attention to the youthful buyers) when buying green items, products, or services. Clearly, green buyers are proficient purchasers, and advertisers have a fundamental need to understand their decisions or choices during their planning to acquire a green item, product, or service. However, green consumption is still developing, and critical research is required for further investigations (Young et al., 2010).

In a broader sense, manageability can be seen as balancing social, natural, and ecological objectives. The most recent perspective on sustainable consumption is that today’s humans ought to secure their needs without trading off future humans’ capacity to obtain their needs. The essential objective of studying sustainable consumption is to focus on buyers who are assumed to have strong convictions and full understandings of sustainability that they apply to their requests and obtaining practices (Schaefer et al., 2005). This knowledge would consequently help makers and advertisers to develop a method by which consumers can acquire their necessities by concentrating on the attitudes, values, sustainable beliefs, and behaviors of buyers.

As far as the market and industry are concerned, consumers’ views on sustainable products are related to different multi-dimensional consumption values. Values assist consumers in making better choices, thereby potentially encouraging green product buying. These values can be emotional, epistemic, social, and functional values (Sheth et al., 1991). In contrast, Sweeney and Soutar (2001) have explained these as functional (including price and quality), emotional, and social values. Tsai (2005) labeled values as affective (emotional and behavioral price), symbolic (reputation), and utilitarian (monetary price and quality). Hedonic value, social value, functional value, and emotional value (Hur et al., 2013) are also highlighted as consumption values.

Values are the dominant consumer factor that encourages sustainable buying behavior. As such, studies show that values play a significant role in consumer buying decisions (Japutra et al., 2021). Accordingly, the present research examines the perception and interconnections regarding consumption value and sustainable consumption behavior in buying or purchasing green products and/or services. Therefore, this work can provide insights to policymakers, green marketers, manufacturers, and suppliers to increase the market share and consumption of eco-friendly sustainable green products and services based on sustainable consumer behavior in South Asia, particularly in developing countries like Pakistan.

The environmentally conscious approach has prompted a sustainable shift in consumer behavior, making individuals aware of their part in achieving sustainability goals (Figueroa-García et al., 2018). Indeed, to better understand the underlying mechanism of sustainable consumption, this study has significantly explored new avenues concerning the factors that strongly predict consumer behavior. Values, beliefs, and attitudes play an essential role in guiding individual behavior. This is a unique study that highlights the significance of six values (functional, social, conditional, environmental, knowledge, and emotional) under the domain of consumers’ behavior.

The rest of the paper is structured as follows. Section 2 presents the theoretical background of this research. The research method is presented in Section 3, followed by the results and discussion in Section 4. Section 5 presents the conclusion.

## Theoretical Background and Hypothesis Development

Nowadays, the term “sustainable” has been related to two different business concepts. Arguably, societies facing ecological crises have regarded environmental concerns as directly influencing consumer buying behavior. Humans have always contributed to developing an effective system for sustaining the land’s natural resources. In recent times, consumers have shown unprecedented interest in organic products, thus drawing the attention of marketers, corporations, and stakeholders toward preserving natural resources (Sdrolia and Zarotiadis, 2019). In this study, defining sustainability includes both components that are involved in sustainable consumer behavior. To enhance understanding of sustainable consumer behavior, a broader view of the concept is required. For that purpose, the literature on applied sustainable business and sustainability should be scrutinized, as the concept of sustainability has acquired significant attention in research.

However, it is difficult to define sustainability (White, 2013). Within research, sustainability has been explained as social equity, environmental stewardship, and equilibrium among financial growth. Another definition arising from the business field, covering numerous essential parts, explains it as “coming across corporate goals and consumer needs in a way that shows the non-stop change for limiting unfavorable effect on individuals and the common habitat” (Duber-Smith, 2010). At present, the research on sustainable consumption behavior is becoming more extensive. However, many studies only focus on purchasing green products in a way that shows environmental awareness (White and Simpson, 2013). Moreover, these studies exclude social equity and financial responsibility. Recently, some studies also cover sustainable consumer behavior from multiple dimensions, recognizing that it goes beyond the implementation scope of green consumption. Also, these studies included the financial and moral responsibility of consumer behavior (Eckhardt et al., 2010; Papaoikonomou, 2013).

Buyers’ perceptions help them decide if an item fits their values regarding the item cost, quality, strength, changelessness, execution, quality, and trustworthiness. These are seen as the primary driving forces for buying green outcome options for customer decision-making behavior (Sheth et al., 1991; Bei and Simpson, 1995). Sheth et al. (1991) characterized functional value (FV) as the apparent efficiency gained through functionality, practicality, or the ability to implement options tangibly. Sweeney and Soutar (2001) saw FV as the effectiveness achieved by the item because this impacts the product’s apparent long and short-term value. It is the value that is assessed later through logical results obtained from the segment of buyers. The buyers incorporate these values to survey numerous cost-effective items supporting the product purchase. It is usually supposed that a sensible consumer intends to attain maximal advantages at the minimal desirable price (Sarfraz et al., 2019; Shah et al., 2019).

Sustainable consumption ensures the integration of the sustainability domains (i.e., social, economic, and environmental) into the product value. The consumers’ perceived product values influence their sustainable behavior, whereas FVs express the product performance in terms of increased functionality (e.g., price, quality, and attributes). However, in terms of product design, the product’s FV increases the likelihood of consumers’ purchase. The prior literature demonstrating the strength of the FV states that buyers’ sustainable consumption largely depends on the product’s FV (Jiang et al., 2022). Indeed, FV plays an essential role in directing the consumer purchase decision.

Furthermore, SV assesses the understanding of the effectiveness extracted from the relationship among social gatherings. Social weight is an imperative driving element of the customer decision process (Sheth et al., 1991; Bei and Simpson, 1995). Thus, the aspect of distinguishing value is explained as a clear advantage of a choice that stems from its image and the symbolic meaning associated with or separated from the convergence of financial, monetary, and socio-cultural references (Connor-Smith and Flachsbart, 2007). Social value suggests a discrete conception regarding what the social order thinks or how it would behave for buying customers. Therefore, policymakers need to understand the direction of social behavior and foresee alterations and changes in individuals’ behavior regarding, for example, energy use (Shove and Walker, 2014; Abdullah et al., 2018).

In recent years, consumers have tended to express themselves as part of society by gaining approval for adopting values that elevate their social acceptance. As a result, this increasing phenomenon makes them involved in purchase behavior that signifies their societal role. Social value is a vehicle for sustainable consumption. For example, consumers buy organic products because of their increased SV. This high social demand encourages individuals to consume the product. Accordingly, studies indicate that SV plays a significant role in promoting sustainable consumer behavior (Wang, 2017).

Conditional value (CV) is expressed by the apparent profit obtained by choosing the final result of a particular situation or a series of conditions that influence decision-makers (Sheth et al., 1991). As per Sánchez-Fernández and Iniesta-Bonillo (2007), “CV refers to the value of a particular product depending on various physical, economic, social, or environmental conditions which may increase the social and functional value of that product.” Biswas and Roy (2015) explained that: “Conditional value is derived as a utility under certain circumstances.” While making a buying decision, every value holds an expected result. The CV is the outcome of different considerations. Indeed, it is a significant factor influencing consumers’ consumption choices. It forms a deep connection with the product characteristics, emphasizing the need to focus on an individual’s buying behavior. CV, referring to the perceived utility of a product, plays a critical role in influencing the product’s sustainable consumption. CV largely escalates the consumer’s buying intention. In explaining this notion, studies demonstrate that CV fosters the demand for green product consumption (Ganak et al., 2020; Shehzad et al., 2020).

Environmental problems that have come to the forefront in the past few decades have given rise to eco-friendly behaviors (Bamberg, 2003). One of the purposes of understanding eco-friendly products is to realize their impact on the environment based on relative assumptions. Further, the objective of eco-friendly products is also to protect the Earth’s environment (Wang et al., 2014). EV involves a range of concepts about environmental problems, like reaching a limited global population, and the connection between environment and growth. Due to the emotional element involved in their view of the issue, customers adjust their utilization techniques and practice environmental safety (Kilbourne and Pickett, 2008). Contemporary views on this topic have led to the New Environmental Paradigm (NEP), which remains the utmost measure of environmentally friendly behavior (Wang et al., 2014). Personal feelings toward the need for natural prevention and a sense of personal duty will activate green buying (Rex and Baumann, 2007).

Values have become the prime driver of today’s consumers’ behavior. Over the last few decades, consumers have been strongly inclined toward buying products that are harmless to the environment. Indeed, this modern consumer ecological belief has made them buy the products that represent their perceived value of the green characteristics. In particular, EV ensures that the product holds the capability to manage environmental issues. In explaining this notion, studies have shown that EV alters consumer behavior by increasing their interest in green products (Tarigan, 2019).

The KVs of an option are characterized as follows: the obvious effectiveness acquired from a choice’s capacity to energize intrigue, give interest, and satisfy a desire for learning data. A choice adds knowledge motivation when consumers study aspects demonstrating elements of premium, peculiarity, and learning. Completely new encounters positively give KVs. For example, a choice that gives a straightforward difference in speed can likewise be permeated with KVs (Carter, 1955). Frequently, environmental information has been the key activator of eco-friendly consumer behavior (Peattie, 2010). Nemcsicsné Zsóka (2008) verified the power of blended observational outcomes to reveal a more intricate relationship between awareness and behavior.

Fryxell and Lo (2003) describe ecological knowledge as the “Personal understanding of the environment, the triggering of key relationships, and the collective responsibility of individuals essential for sustainable development.” Though environmental problems are considered, environmental knowledge alters environmental attitudes, and individuals’ purchasing behavior is further affected by environmental knowledge (Scott et al., 2014). Accordingly, the consumers contribute to sustainable development through their buying choices. The cognitive element of KV rationally assists the consumer buying behavior. The KV provides the relevant information to the individual regarding product use, attributes, and functionality, thus encouraging sustainable consumption behavior. This insight allows the consumers to base their decision on knowledge objectively, thus following the long-standing tradition of consumption patterns (Rizkalla, 2018).

**FIGURE 1:**
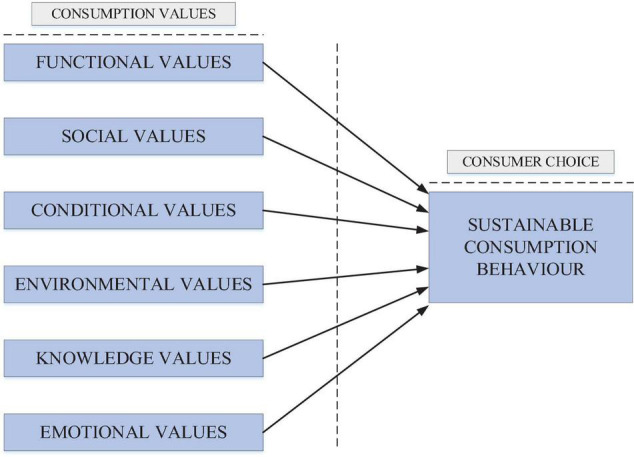
Study framework.

Emotional values are the outward expressions that demonstrate a personal capacity to excite sentiments before sensation conditions (Sheth et al., 1991). Merchandise and ventures are often strongly related to emotional reactions. In contrast to the other measures, it incorporates serviceable and indulgent segments (Sweeney and Soutar, 2001). The significance of this aspect is evident in a remark by MacKay et al. (1999). According to Mackay, the interest in an item or an administration is the combination of normal and enthusiastic elements, and that feeling influences each buying choice. The consumer, believing that their activities influence the market activities, make choices based on the EMV. In particular, the study shows that EMVs manifest in the consumers’ experience, thus ensuring sustainable consumption behavior (Khan and Mohsin, 2017). As per Hartmann et al. (2005), once the buyer is purchasing green items, the impact of EMV is substantially more grounded than FV advantages. Due to utilizing green product items, customers feel warm prosperity because of the ethical fulfillment achieved by helping Earth. Hence, understanding this relationship provides critical insights to the marketers to emphasize the EMV of the green purchase (Joshi et al., 2021). Figure 1 shows the study framework.

In this study, we have developed six hypotheses:

H1: Function values significantly influence sustainable consumption behavior.

H2: Social values significantly influence sustainable consumption behavior.

H3: Conditional values significantly influence sustainable consumption behavior.

H4: Environmental values significantly influence sustainable consumption behavior.

H5: Knowledge values significantly influence sustainable consumption behavior.

H6: Emotional values significantly influence sustainable consumption behavior.

## Research Methodology

This study determines consumer value factors and SCB to measure consumers’ choice behaviors for eco-friendly products. This section explains the surveyed area, data sources, methodology, data collection tool, questionnaire, research model/framework, dependent variable, and independent variables. The survey was conducted in two different countries: Pakistan and China. In Pakistan, the educational institutions located in Khyber Pakhtunkhwa province were considered. In China, Chongqing city, which is located in one of the four municipalities of China, named Chongqing, was considered.

Primary quantitative data were gathered from the sources. Data were collected firsthand through a survey questionnaire source. The sample for the research purpose was selected during the period from June 6, 2021 to June 28, 2021 in China and from August 23, 2021 to October 5, 2021 in Pakistan. The research population of this study includes consumers from Khyber Pakhtunkhwa, Pakistan, and Chongqing, China. Random sampling was used for the collection and analysis of the data. A cross-sectional study was done. The size of the study sample included in this research was 431 respondents from different universities in Abbottabad, including COMSATS University Islamabad, Abbottabad Campus, Frontier Medical College, and Women’s Medical College, and 342 respondents from the universities located in Chongqing, China. The data were gathered by a survey using a questionnaire. The anonymity and confidentiality of the respondents were assured, and the data were used only for academic purposes. The demographic information of the respondents from Pakistan and China is summarized in Tables 1, 2. In Pakistan, among the 431 valid responses, 229 (53.1%) were men, and the rest 202 (46.9%) were women. There were 342 valid responses from China; among them, 177 (51.8%) were men, and 165 (48.2%) were women, which was close to the ideal 1: 1 ratio.

**TABLE 1:** Profile of respondents from Pakistan.

Variables	Options	Frequency	Percent	Valid percent	Cumulative percent
Gender	Male	229	53.1	53.1	53.1
	Female	202	46.9	46.9	100
	Total	431	100	100	
Age	16–20	135	31.3	31.3	31.3
	21–25	236	54.8	54.8	86.1
	26–30	43	10.0	10.0	96.1
	31–35	9	2.1	2.1	98.1
	36–40	2	0.5	0.5	98.6
	41–45	4	0.9	0.9	99.5
	46–50	2	0.5	0.5	100.0
	Total	431	100.0	100.0	
Marital status	Married	74	17.2	17.2	17.2
	Unmarried	357	82.8	82.8	100.0
	Total	431	100.0	100.0	
Education	Bachelors	280	65.0	65.0	65.0
	Masters	135	31.3	31.3	96.3
	Ph.D.	16	3.7	3.7	100.0
	Total	431	100.0	100.0	
Family income	Below 50,000	94	21.8	21.8	21.8
	50,001–70,000	109	25.3	25.3	47.1
	70,001–90,000	77	17.9	17.9	65.0
	90,001–110,000	53	12.3	12.3	77.3
	110,001–130,000	26	6.0	6.0	83.3
	130,001–150,000	22	5.1	5.1	88.4
	150,001–200,000	12	2.8	2.8	91.2
	Above 200,000	38	8.8	8.8	100.0
	Total	431	100.0	100.0	

**TABLE 2:** Profile of respondents from China.

Variables	Options	Frequency	Percent	Valid percent	Cumulative percent
Gender	Male	177	51.8	51.8	51.8
	Female	165	48.2	48.2	100.0
	Total	342	100.0	100.0	
Age	16–20	81	23.7	23.7	23.7
	21–25	182	53.2	53.2	76.9
	26–30	44	12.9	12.9	89.8
	31–35	17	5.0	5.0	94.7
	36–40	10	2.9	2.9	97.7
	41–45	4	1.2	1.2	98.8
	46–50	2	0.6	0.6	99.4
	51–55	2	0.6	0.6	100.0
	Total	342	100.0	100.0	
Marital status	Married	57	16.7	16.7	16.7
	Unmarried	285	83.3	83.3	100.0
	Total	342	100.0	100.0	
Education	Bachelors	191	55.8	55.8	55.8
	Masters	119	34.8	34.8	90.6
	Ph.D.	32	9.4	9.4	100.0
	Total	342	100.0	100.0	
Family income	0–5,000	263	76.9	76.9	76.9
	5,001–10,000	62	18.1	18.1	95.0
	10,001–20,000	12	3.5	3.5	98.5
	20,001–30,000	1	0.3	0.3	98.8
	30,001–50,000	4	1.2	1.2	100.0
	Total	342	100.0	100.0	

### Data Collection

For the collection of data, a structured questionnaire was used. The instruments in the survey questionnaire were divided into three parts: consumer demographic characteristics, consumption values, and sustainable consumption behavior to evaluate consumer choice behavior regarding eco-friendly products. A five-point Likert scale was used, ranging from 1 to 5 to show the level of disagreement or agreement (1 = Strongly disagree, 2 = Disagree, 3 = Neutral, 4 = Agree, and 5 = Strongly agree) (Lin and Huang, 2012; Wang et al., 2014). The six elements of the questionnaire measure the scale of sustainable consumption behavior (Wang et al., 2014). The six consumption values scales were measured by 22 items (Lin and Huang, 2012). Table 1 shows the profile of respondents from Pakistan, while Table 2 represents the profile of respondents from China.

## Results

Before testing the model and hypotheses, reliability tests were performed on each constructor variable. Cronbach’s alpha results (Table 3) confirm that the reliability statistics range from 0.683 to 0.771; therefore, it is in the range (essentially, 0.61–0.80) and more stringent (almost perfect, 0.81–1.0) (Landis and Koch, 1977). Therefore, the reliability of the questionnaire is quite very high and is verified. Regarding the sample’s representativeness, the systematic difference between 431 and 342 respondents was evaluated using the sample mean difference test.

**TABLE 3:** Reliability analysis.

Constructs/variables	No. of items	Cronbach’s alpha (Pakistan)	Cronbach’s alpha (China)
Functional value	3	0.689	0.650
Social value	4	0.690	0.704
Conditional value	5	0.746	0.768
Environmental value	4	0.771	0.856
Knowledge value	3	0.683	0.782
Emotional value	3	0.762	0.844
Choice behavior	4	0.709	0.783
Overall	26	0.927	0.916

In Table 4, Levene’s test for variance between the two groups shows that the homogeneity of variance assumption is not violated. Due to the consistency of the hypothesis, the main results indicate that there is no significant difference in the average value at a p-value less than 0.05.

**TABLE 4:** Independent samples *t*-test.

		Levene’s test for equality of variances		*t*-test for equality of means	
		*F*	Sig.	*t*	Sig. (2-tailed)
FV	Equal variances assumed	5.523	0.019	−2.192	0.029
	Equal variances not assumed			−2.231	0.026
SV	Equal variances assumed	5.745	0.017	−4.007	0.000
	Equal variances not assumed			−4.065	0.000
CV	Equal variances assumed	17.803	0.000	−7.394	0.000
	Equal variances not assumed			−7.522	0.000
EV	Equal variances assumed	15.314	0.000	−7.639	0.000
	Equal variances not assumed			−7.764	0.000
KV	Equal variances assumed	0.414	0.520	−4.785	0.000
	Equal variances not assumed			−4.799	0.000
EMV	Equal variances assumed	6.018	0.014	−1.194	0.233
	Equal variances not assumed			−1.208	0.227
CVB	Equal variances assumed	15.171	0.000	−2.413	0.016
	Equal variances not assumed			−2.465	0.014

### Descriptive Statistics

Table 5 shows the average mean score and standard deviation of the variables in the study conducted in Pakistan, respectively. Table 6 presents the descriptive statistics of variables in the study conducted in China. The instruments for testing the consumer’s response agreement use a format scale, and the results have determined a mutual level of agreement. It discloses the level of consumers’ agreement with all variables and the statements about their traditional activities. Starting with the consumer’s response to green products and services out of a maximum score of 5, most mean score values were above 3.30. The results demonstrate that our sample has favorable estimations or suitable appraisals about green products or services. Consumers believe that buying or purchasing eco-friendly green products is easy and under their control.

**TABLE 5:** Descriptive statistics of variables in the study conducted in Pakistan.

Constructs/variables	*N*	Minimum	Maximum	Mean	Std. deviation
FV	431	1.00	5.00	3.3020	0.88301
SV	431	1.00	5.00	3.4132	0.83201
CV	431	1.00	5.00	3.4608	0.82701
EV	431	1.00	5.00	3.6224	0.95079
KV	431	1.00	5.00	3.4395	0.89848
EMV	431	1.00	5.00	3.6620	0.97288
CVB	431	1.00	5.00	3.4377	0.90129

**TABLE 6:** Descriptive statistics of variables in the study conducted in China.

Constructs/variables	*N*	Minimum	Maximum	Mean	Std. deviation
FV	342	1.00	5.00	3.4337	0.75739
SV	342	1.00	5.00	3.6425	0.73449
CV	342	1.00	5.00	3.8773	0.71131
EV	342	1.00	5.00	4.1187	0.82461
KV	342	1.00	5.00	3.7476	0.87568
EMV	342	1.00	5.00	3.7427	0.87881
CVB	342	1.00	5.00	3.5851	0.75536

### Correlation Results

The Pearson correlation test was employed to check the correlation between all variables. The six consumer value scales were positively and significantly correlated with consumer choice behavior (*P* < 0.01). Table 7 shows the correlation results for Pakistan, where the highest observed coefficient is 0.622, which exists between CV and EV indices. The SV is positively correlated with CV (0.607). The lowest positive correlation occurs between FV and KV. Table 8 shows correlation results for China, where the highest observed coefficient is 0.598, and it exists between CV and SV. The EMV is positively correlated with CV (0.593). The lowest positive correlation occurs between EV and FV and between CVB and FV.

**TABLE 7:** Correlation among variables (Pakistan).

	FV	SV	CV	EV	KV	EMV	CVB
FV	1						
SV	0.529**	1					
CV	0.475**	0.607**	1				
EV	0.429**	0.564**	0.622**	1			
KV	0.337**	0.495**	0.527**	0.539**	1		
EMV	0.395**	0.593**	0.580**	0.605**	0.529**	1	
CVB	0.385**	0.449**	0.460**	0.480**	0.540**	0.568**	1

**Correlation is significant at the 0.01 level (two-tailed).

**TABLE 8:** Correlation among variables (China).

	FV	SV	CV	EV	KV	EMV	CVB
FV	1						
SV	0.458**	1					
CV	0.435**	0.598**	1				
EV	0.284**	0.311**	0.536**	1			
KV	0.290**	0.359**	0.473**	0.467**	1		
EMV	0.406**	0.566**	0.593**	0.484**	0.462**	1	
CVB	0.284**	0.398**	0.474**	0.423**	0.489**	0.504**	1

**Correlation is significant at the 0.01 level (two-tailed).

### Influence of Consumption Values on Sustainable Consumption Behavior

Sustainable consumer behavior was computed based on all the consumption values of the 431 and 342 respondents from Pakistan and China, respectively. The six variables entered in the model for Pakistan with an EMV variance of 94.6%, followed by EV (90.4%), KV (80.7%), FV (78.0%), SV (69.2%), and CV (68.4%), whereas the variance values for China were KV (77.4%), EMV (77%), EV (68.0%), FV (57.3%), CVB (57.1%), SV (54.4%), and CV (51.1%). By calculating the acceptability of all variables and the variance inflationary factor (VIF), the existence of collinearity in the model was verified. The low variance inflation factor (less than 2.2) and VIF value less than 5 indicate no multicollinearity problems in the model.

A multiple linear regression analysis was carried out with the choice behavior (sustainable consumption behavior) as the dependent variable and consumption values (FV, SV, CV, EV, KV, and EMV) as the independent variables. The results in Tables 9, 10 reveal that the KV and EMV have a positive and significant influence on the respondent choice behavior (sustainable consumption behavior) regarding environmentally friendly green products in both countries. In contrast, CV and SV do not significantly influence sustainable consumption behavior.

**TABLE 9:** Regression results among the variables (Pakistan).

Model	Unstandardized coefficients		*t*	Sig.	Collinearity statistics	
	Beta	Std. error			Tolerance	VIF
(Constant)	0.609	0.172	3.538	0.000		
FV	0.122	0.046	2.676	0.008	0.677	1.477
SV	0.021	0.058	0.356	0.722	0.478	2.094
CV	0.040	0.059	0.682	0.495	0.472	2.121
EV	0.075	0.050	1.492	0.136	0.485	2.064
KV	0.273	0.047	5.748	0.000	0.605	1.652
EMV	0.274	0.048	5.676	0.000	0.498	2.007
**a. Dependent variable: CVB**						
**Test statistics**						
*R* ^2^	0.426					
Adjusted *R*^2^	0.418					
*F*-statistics	52.389					
Sig. value	0.000					

**TABLE 10:** Regression results among the variables (China).

Model	Unstandardized coefficients		*t*	Sig.	Collinearity statistics	
	Beta	Std. error			Tolerance	VIF
(Constant)	0.879	0.221	3.973	0.000		
FV	0.003	0.051	0.066	0.948	0.735	1.360
SV	0.079	0.061	1.290	0.198	0.538	1.859
CV	0.126	0.068	1.845	0.066	0.455	2.196
EV	0.099	0.051	1.945	0.053	0.617	1.620
KV	0.216	0.046	4.732	0.000	0.677	1.476
EMV	0.187	0.052	3.573	0.000	0.515	1.943
**a. Dependent variable: CVB**						
**Test statistics**						
*R* ^2^	0.367					
Adjusted *R*^2^	0.356					
*F*-statistics	31.981					
Sig. value	0.000					

Additionally, FV and EV show contrary results in both countries. FV has a significant influence in Pakistan, whereas it is insignificant in China, while EV has opposite influences in both countries concerning sustainable consumption behavior (see Table 11).

**TABLE 11:** Comparison of variables between Pakistan and China.

	Knowledge Values (KVs)	Emotional Values (EMVs)	Functional Values (FVs)	Environmental Values (EVs)	Social Values (SVs)	Conditional Values (CVs)
Pakistan	Sig.*	Sig.*	Sig.*	Insig.**	Insig.**	Insig.**
China	Sig.*	Sig.*	Insig.**	Sig.*	Insig.**	Insig.**

*Sig., Significant; **Insig., Insignificant.

If the consumers in Pakistan attach higher FV, KV, and EMV to eco-friendly products, the probability of the consumers selecting these products will be high, supporting H1, H5, and H6. Consumers in China show an inclination toward EV, KV, and EMV, supporting H4, H5, and H6.

## Discussion

Over the years, environmental concerns have become more prevalent due to the world’s accelerating consumption rate. The issue of ecological damage has become critical because of the increased consumption of natural resources. In managing this situation, values have emerged as the prime determinant driving sustainable consumer behavior (Orellano et al., 2020). Indeed, the environmental aspect has seen consumers contribute toward sustainable development by massively increasing the demand for eco-friendly products.

The KV of environmentally friendly products has a significant and positive effect on consumer choice behavior, leading consumers to have a thirst for knowledge, or fondness of uniqueness, regarding environmentally friendly products. According to Lin and Huang (2012), product makers expecting to build buyer knowledge and awareness can consider the attributes of their items, products, or services, and make efforts to outline the standards and benefits of green eco-friendly products. Indeed, the literature shows that the perception of non-toxic products (i.e., natural and pollutant-free) increases the individual’s belief in the product characteristics, thereby positively stimulating sustainable buying (Jaiswal and Kant, 2018).

Moreover, EMV expresses the buyers’ connectivity with the brand. EMV establishes a strong customer–product bonding, thereby influencing the consumers’ purchase choices. EMV also has a significant effect on consumer choice behavior. Hence, the consumption value theory indicates a positive relationship between EMV and consumer choice behavior (Khan and Mohsin, 2017). Additionally, SV, as applied to the sustainability frame, strongly influences consumers’ buying behavior. Notably, a few respondents did not feel that practicing environmental awareness expands social endorsement or establishes a decent connection. Even when people are very concerned about nature and the environment, they still strongly believe that protecting the environment is the responsibility of the government and large companies (Laroche et al., 2001). For example, research by Kalafatis et al. (1999) regarding customer behavior in Greece demonstrated that buyers base their activities more on individual conviction rather than on social weight.

The conditional variable is a variable that does not affect consumer choice behavior in both countries studied herein; it does not have an imperative association with green product qualities. There are two approaches related to this value. The first is the greenhouse effect, which poses a huge threat to our natural environment. Second, governments and green groups need to stress the implications of climatic change and how it relates to the natural environmental burden (Biswas and Roy, 2015). Situational factors suggest the conditions surrounding the people impact their reactions to improvements relevant to their requirements and desires. One study, for example, found that travel times to the marketplace in India strongly influenced the buying of different items or products, while travel time to the shopping center in the United States was insignificant regarding the purchase of foodstuffs, beverages, or other different products. As stated by Sánchez-Fernández and Iniesta-Bonillo (2007), the CV indicates that certain market choices depend on the circumstances or conditions provided to the buyer.

Moreover, a product’s FV is a strong predictor of consumer behavior. Consumers perceiving high FV are encouraged toward sustainable buying behavior. Based on this, the literature states that FV increases the demand for the products, thereby influencing consumers’ sustainable consumption (Park and Lin, 2020). Accordingly, the aftereffects of multiple regression analysis indicate that FV and cost seem to impact Pakistani buyers’ purchase behavior significantly. Laroche et al. (2001) conducted several studies on sustainable and green consumption, and the results indicate that people are usually and progressively willing to pay more for green products and services. This behavior demonstrates the desire to exchange, where the factors and conditions of the green product are good for the environment in a way that exceeds the cost factors. In this way, FV, cost, and the nature of green products and services are key variables impacting consumers’ sustainable consumption behavior. The results of China reveal that quality and price have no significant impact on consumer choice behavior.

Consumers who are highly concerned about the environment have more confidence in the price and quality and have more positive attitudes toward eco-friendly products and services. Compared with consumers with lower environmental and natural values, consumers with higher ecological concerns may be concerned about discovering improved environmental friendliness and related social incentives.

When faced with certain situations or circumstances, such as declining natural environments or the accessibility of products or rebates for eco-friendly items and products, buyers with more environmental natural values are eager to become more environmentally friendly. Such purchasers, moreover, are extra influential in seeking information after buying green product items and are more likely to pursue uniqueness. Thus, the more the customers are worried about the environment, the more they are encouraged to buy green products (Lin and Huang, 2012).

## Conclusion

This manuscript establishes a framework for discovering green consumer behavior in Pakistan and China, examining the associations between consumption values and sustainable consumption behavior. Multiple linear regression and correlation analysis were employed to clarify the influence factors on green consumer behavior regarding different consumption values. The outcomes reveal variations among choice behaviors for independent variables. In Pakistan, consumer choice behavior showed a significant relationship with FVs, KVs, and EMVs, whereas SVs, CVs, and EVs were insignificant. For China, the EVs, KVs, and EMVs turned out to be significant, whereas FVs, SVs, and CVs values show insignificant influence on sustainable consumption behavior.

The KV and EMV of eco-friendly products significantly influence consumer choice behavior in both Asian countries. The KV brings in consumers through an interest, desire for knowledge, or fondness for uniqueness. With the EMV, green research marketing realizes the buyers’ feelings and emotions toward the ecological concern, which triggers their choices to consume green products. The FV, which was found to be significant in Pakistan, has directed various research studies on sustainable consumption. Notably, all the findings exhibit the notion that individuals are progressively ready to spend more on eco-friendly items. The Chinese customers give more importance to EVs; China has acknowledged a particular aspect of natural effect and has applied this to upcoming advancements.

Government policies and instructive educational agendas are essential to encourage and change customer choices toward environmentally friendly products and services. In detailing these projects, strategy makers need to consider that growing the trust level of consumers in this capacity is crucial.

## Data Availability Statement

The raw data supporting the conclusions of this article will be made available by the authors, without undue reservation.

## Ethics Statement

Ethical review and approval was not required for the study on human participants in accordance with the local legislation and institutional requirements. The patients/participants provided their written informed consent to participate in this study.

## Author Contributions

All authors listed have made a substantial, direct, and intellectual contribution to the work, and approved it for publication.

## Conflict of Interest

The authors declare that the research was conducted in the absence of any commercial or financial relationships that could be construed as a potential conflict of interest.

## Publisher’s Note

All claims expressed in this article are solely those of the authors and do not necessarily represent those of their affiliated organizations, or those of the publisher, the editors and the reviewers. Any product that may be evaluated in this article, or claim that may be made by its manufacturer, is not guaranteed or endorsed by the publisher.

## References

[B1] Abdullah M. I. Sarfraz M. Arif A. Azam A. (2018). An extension of the theory of planned behavior towards brand equity and premium price. *Pol. J. Manage. Stud.* 18 20–32.

[B2] Ajzen I. (1991). The theory of planned behavior. *Organ. Behav. Hum. Decis. Process.* 50 179–211.

[B3] Ajzen I. Fishbein M. (1980). *Understanding Attitudes and Predicting Social Behavior.* Hoboken, NJ: Prentice-Hall.

[B4] Bamberg S. (2003). How does environmental concern influence specific environmentally related behaviors? A new answer to an old question. *J. Environ. Psychol.* 23 21–32. 10.1016/S0272-4944(02)00078-6

[B5] Bei L.-T. Simpson M. E. (1995). The determinants of consumers’ purchase decisions for recycled products: an application of acquisition-transaction utility theory. *Adv. Consum. Res.* 22 257–261. 10.3390/ijerph17186607 32932797 PMC7559813

[B6] Biswas A. Roy M. (2015). Green products: an exploratory study on the consumer behaviour in emerging economies of the East. *J. Clean. Prod.* 87 463–468. 10.1016/j.jclepro.2014.09.075

[B7] Brach S. Walsh G. Shaw D. (2018). Sustainable consumption and third-party certification labels: consumers’ perceptions and reactions. *Eur. Manage. J.* 36 254–265. 10.1016/j.emj.2017.03.005

[B8] Carter R. E. (1955). Katz and Lazarsfeld. Personal influence: the part played by people in the flow of mass communications (book review). *Soc. Forces* 34:383. 10.2307/2573681

[B9] Chan R. Y. K. (2001). Determinants of Chinese consumers’ green purchase behavior. *Psychol. Mark.* 18 389–413.

[B10] Connor-Smith J. K. Flachsbart C. (2007). Relations between personality and coping: a meta-analysis. *J. Pers. Soc. Psychol.* 93 1080–107. 10.1037/0022-3514.93.6.1080 18072856

[B11] Diamantopoulos A. Schlegelmilch B. B. Sinkovics R. R. Bohlen G. M. (2003). Can socio-demographics still play a role in profiling green consumers? A review of the evidence and an empirical investigation. *J. Bus. Res.* 56 465–480.

[B12] Duber-Smith D. (2010). What to do about sustainability-darrin duber-smith explains why it is important for companies to develop a plan, add natural and certified organic ingredients in stages, and communicate to the consumer in a transparent and effective manner. *Household Pers. Products Ind.* 47:42.

[B13] Eckhardt G. M. Belk R. Devinney T. M. (2010). Why don’t consumers consume ethically? *J. Consum. Behav.* 9 426–436. 10.1002/cb.332

[B14] Figueroa-García E. García-Machado J. Pérez-Bustamante Yábar D. (2018). Modeling the social factors that determine sustainable consumption behavior in the community of Madrid. *Sustainability* 10:2811. 10.3390/su10082811

[B15] Fryxell G. E. Lo C. W.-H. (2003). The influence of environmental knowledge and values on managerial behaviours on behalf of the environment: an empirical examination of managers in China. *J. Bus. Ethics* 46 45–69. 10.1023/A:1024773012398

[B16] Ganak J. Chen Y. Liang D. Liu H. Chi T. (2020). Understanding US millennials’ perceived values of denim apparel recycling: insights for brands and retailers. *Int. J. Sustain. Soc.* 12 267–290. 10.1504/IJSSOC.2020.112444

[B17] Hartmann P. Apaolaza Ibáñez V. Forcada Sainz F. J. (2005). Green branding effects on attitude: functional versus emotional positioning strategies. *Mark. Intell. Plan.* 23 9–29. 10.1108/02634500510577447

[B18] Hur W.-M. Kim Y. Park K. (2013). Assessing the effects of perceived value and satisfaction on customer loyalty: a ‘green’ perspective. *Corp. Soc. Responsib. Environ. Manage.* 20 146–156. 10.1002/csr.1280

[B19] Jaiswal D. Kant R. (2018). Green purchasing behaviour: a conceptual framework and empirical investigation of Indian consumers. *J. Retail. Consum. Serv.* 41 60–69. 10.1016/j.jretconser.2017.11.008

[B20] Jang Y. J. Kim W. G. Bonn M. A. (2011). Generation Y consumers’ selection attributes and behavioral intentions concerning green restaurants. *Int. J. Hosp. Manag.* 30 803–811. 10.1016/j.ijhm.2010.12.012

[B21] Japutra A. Loureiro S. M. C. Wang S. (2021). The role of personal values and personality traits on intention to recommend a destination. *Tourism Anal.* 26 349–361. 10.3727/108354220X15987151867872

[B22] Jiang S. Jotikasthira N. Pu R. (2022). Toward sustainable consumption behavior in online education industry: the role of consumer value and social identity. *Front. Psychol.* 13:865149. 10.3389/fpsyg.2022.865149 35465533 PMC9022665

[B23] Joshi Y. Uniyal D. P. Sangroya D. (2021). Investigating consumers’ green purchase intention: examining the role of economic value, emotional value and perceived marketplace influence. *J. Clean. Prod.* 328:129638. 10.1016/j.jclepro.2021.129638

[B24] Kalafatis S. P. Pollard M. East R. Tsogas M. H. (1999). Green marketing and Ajzen’s theory of planned behaviour: a cross-market examination. *J. Consum. Mark.* 16 441–460. 10.1108/07363769910289550

[B25] Khan S. N. Mohsin M. (2017). The power of emotional value: exploring the effects of values on green product consumer choice behavior. *J. Clean. Prod.* 150 65–74. 10.1016/j.jclepro.2017.02.187

[B26] Khare A. (2015). Antecedents to green buying behaviour: a study on consumers in an emerging economy. *Mark. Intell. Plan.* 33 309–329. 10.1108/MIP-05-2014-0083

[B27] Kilbourne W. Pickett G. (2008). How materialism affects environmental beliefs, concern, and environmentally responsible behavior. *J. Bus. Res.* 61 885–893. 10.1016/j.jbusres.2007.09.016

[B28] Kim Y. J. Njite D. Hancer M. (2013). Anticipated emotion in consumers’ intentions to select eco-friendly restaurants: augmenting the theory of planned behavior. *Int. J. Hosp. Manag.* 34 255–262. 10.1016/j.ijhm.2013.04.004

[B29] Landis J. R. Koch G. G. (1977). The measurement of observer agreement for categorical data. *Biometrics* 33 159–74. 10.2307/2529310 843571

[B30] Laroche M. Bergeron J. Barbaro-Forleo G. (2001). Targeting consumers who are willing to pay more for environmentally friendly products. *J. Consum. Mark.* 18 503–520. 10.1108/EUM0000000006155

[B31] Leonidou C. N. Katsikeas C. S. Morgan N. A. (2013). “Greening” the marketing mix: do firms do it and does it pay off? *J. Acad. Mark. Sci.* 41 151–170. 10.1007/s11747-012-0317-2

[B32] Lin P.-C. Huang Y.-H. (2012). The influence factors on choice behavior regarding green products based on the theory of consumption values. *J. Clean. Prod.* 22 11–18. 10.1016/j.jclepro.2011.10.002

[B33] MacKay D. G. Abrams L. Pedroza M. J. (1999). Aging on the input versus output side: theoretical implications of age-linked asymmetries between detecting versus retrieving orthographic information. *Psychol. Aging* 14 3–17. 10.1037//0882-7974.14.1.3 10224628

[B34] Mendelson B. K. White D. R. Mendelson M. J. (1996). Self-esteem and body esteem: effects of gender, age, and weight. *J. Appl. Dev. Psychol.* 17 321–346.

[B35] Moisander J. (2007). Motivational complexity of green consumerism. *Int. J. Consum. Stud.* 31 404–409. 10.1111/j.1470-6431.2007.00586.x

[B36] Moser A. K. (2015). Thinking green, buying green? Drivers of pro-environmental purchasing behavior. *J. Consum. Mark.* 32 167–175. 10.1108/JCM-10-2014-1179

[B37] Nemcsicsné Zsóka Á (2008). Consistency and “awareness gaps” in the environmental behaviour of Hungarian companies. *J. Clean. Prod.* 16 322–329. 10.1016/j.jclepro.2006.07.044

[B38] Orellano A. Valor C. Chuvieco E. (2020). The influence of religion on sustainable consumption: a systematic review and future research agenda. *Sustainability* 12:7901. 10.3390/su12197901

[B39] Papaoikonomou E. (2013). Sustainable lifestyles in an urban context: towards a holistic understanding of ethical consumer behaviours. Empirical evidence from Catalonia, Spain. *Int. J. Consum. Stud.* 37 181–188. 10.1111/j.1470-6431.2012.01111.x

[B40] Park H. J. Lin L. M. (2020). Exploring attitude–behavior gap in sustainable consumption: comparison of recycled and upcycled fashion products. *J. Bus. Res.* 117 623–628. 10.1016/j.jbusres.2018.08.025

[B41] Peattie K. (2010). Green consumption: behavior and norms. *Ann. Rev. Environ. Resour.* 35 195–228. 10.1146/annurev-environ-032609-094328

[B42] Pino G. Peluso A. M. Guido G. (2012). Determinants of regular and occasional consumers’ intentions to buy organic food. *J. Consum. Affairs* 46 157–169.

[B43] Raghavan R. Bright C. L. Shadoin A. L. (2008). Toward a policy ecology of implementation of evidence-based practices in public mental health settings. *Implement. Sci.* 3:26. 10.1186/1748-5908-3-26 18485219 PMC2396668

[B44] Ramayah T. Lee J. W. C. Mohamad O. (2010). Green product purchase intention: some insights from a developing country. *Resour. Conserv. Recycling* 54 1419–1427. 10.1016/j.resconrec.2010.06.007

[B45] Rex E. Baumann H. (2007). Beyond ecolabels: what green marketing can learn from conventional marketing. *J. Clean. Prod.* 15 567–576. 10.1016/j.jclepro.2006.05.013

[B46] Rizkalla N. (2018). Determinants of sustainable consumption behavior: an examination of consumption values, PCE environmental concern and environmental knowledge. *Int. J. Soc. Sci. Hum.* 7 48–54. 10.18178/ijssh.2018.V8.932

[B47] Rusyani E. Lavuri R. Gunardi A. (2021). Purchasing eco-sustainable products: interrelationship between environmental knowledge, environmental concern, green attitude, and perceived behavior. *Sustainability* 13:4601. 10.3390/su13094601

[B48] Rylander D. Allen C. (2001). Understanding green consumption behavior: toward an integrative framework. *Am. Mark. Assoc. Winter Educ. Conf. Proc.* 11 386–387.

[B49] Sánchez-Fernández R. Iniesta-Bonillo M. Á (2007). The concept of perceived value: a systematic review of the research. *Mark. Theory* 7 427–451. 10.1177/1470593107083165

[B50] Sarfraz M. Qun W. Shah S. G. M. Fareed Z. (2019). Do hierarchical jumps in CEO succession invigorate innovation? Evidence from Chinese economy. *Sustainability* 11:2017. 10.3390/su11072017

[B51] Schaefer A. M. Sanes J. R. Lichtman J. W. (2005). A compensatory subpopulation of motor neurons in a mouse model of amyotrophic lateral sclerosis. *J. Comp. Neurol.* 490 209–219. 10.1002/cne.20620 16082680

[B52] Schuhwerk M. E. Lefkoff-Hagius R. (1995). Green or non-green? does type of appeal matter when advertising a green product? *J. Advert.* 24 45–54. 10.1080/00913367.1995.10673475

[B53] Scott I. M. Clark A. P. Josephson S. C. Boyette A. H. Cuthill I. C. Fried R. L. (2014). Human preferences for sexually dimorphic faces may be evolutionarily novel. *Proc. Natl. Acad. Sci. U. S. A.* 111 14388–14393. 10.1073/pnas.1409643111 25246593 PMC4210032

[B54] Sdrolia E. Zarotiadis G. (2019). A comprehensive review for green product term: from definition to valuation. *J. Econ. Surveys* 33 150–178. 10.1111/joes.12268

[B55] Shah S. G. M. Sarfraz M. Fareed Z. Rehman M. A. Maqbool A. Qureshi M. A. A. (2019). Whether CEO succession *via* hierarchical jumps is detrimental or blessing in disguise? evidence from Chinese listed firms. *Zagreb Int. Rev. Econ. Bus.* 22 23–41. 10.2478/zireb-2019-0018

[B56] Shehzad K. Xiaoxing L. Sarfraz M. Zulfiqar M. (2020). Signifying the imperative nexus between climate change and information and communication technology development: a case from Pakistan. *Environ. Sci. Pollut. Res.* 27 30502–30517. 10.1007/s11356-020-09128-x 32468367

[B57] Sheth J. N. Newman B. I. Gross B. L. (1991). Why we buy what we buy: a theory of consumption values. *J. Bus. Res.* 22 159–170. 10.1016/0148-2963(91)90050-8

[B58] Shove E. Walker G. (2014). What is energy for? social practice and energy demand. *Theory Cult. Soc.* 31 41–58. 10.1177/0263276414536746

[B59] Sweeney J. C. Soutar G. N. (2001). Consumer perceived value: the development of a multiple item scale. *J. Retail.* 77 203–220. 10.1016/S0022-4359(01)00041-0

[B60] Tarigan A. K. M. (2019). Expectations, attitudes, and preferences regarding support and purchase of eco-friendly fuel vehicles. *J. Clean. Prod.* 227 10–19. 10.1016/j.jclepro.2019.04.190

[B61] Tsai S. (2005). Impact of personal orientation on luxury-brand purchase value: an international investigation. *Int. J. Mark. Res.* 47 427–452. 10.1177/147078530504700403

[B62] Wang P. Liu Q. Qi Y. (2014). Factors influencing sustainable consumption behaviors: a survey of the rural residents in China. *J. Clean. Prod.* 63 152–165. 10.1016/j.jclepro.2013.05.007

[B63] Wang T. (2017). Social identity dimensions and consumer behavior in social media. *Asia Pac. Manag. Rev.* 22 45–51. 10.1016/j.apmrv.2016.10.003

[B64] White K. Simpson B. (2013). When do (and don’t) normative appeals influence sustainable consumer behaviors? *J. Mark.* 77 78–95.

[B65] White M. A. (2013). Sustainability: I know it when I see it. *Ecol. Econ.* 86 213–217. 10.1016/j.ecolecon.2012.12.020

[B66] Young W. Hwang K. McDonald S. Oates C. J. (2010). Sustainable consumption: green consumer behaviour when purchasing products. *Sustain. Dev.* 18 20–31. 10.1002/sd.394

